# Evaluation of Aminolevulinic Acid-Derived Tumor Fluorescence Yields Disparate Results in Murine and Spontaneous Large Animal Models of Lung Cancer

**DOI:** 10.1038/s41598-019-40334-x

**Published:** 2019-05-21

**Authors:** Jarrod D. Predina, Jeffrey Runge, Andrew Newton, Michael Mison, Leilei Xia, Christopher Corbett, Michael Shin, Lydia Frenzel Sulyok, Amy Durham, Shuming Nie, Sunil Singhal, David Holt

**Affiliations:** 1 Center for Precision Surgery, Perelman School of Medicine at the University of Pennsylvania, Philadelphia, USA; 2 Department of Clinical Studies, University of Pennsylvania School of Veterinary Medicine, Philadelphia, USA; 3 Department of Pathobiology, University of Pennsylvania School of Veterinary Medicine, Philadelphia, USA; 4 Departments of Biomedical Engineering and Chemistry, Emory University, Atlanta, Georgia

**Keywords:** Molecular medicine, Surgical oncology

## Abstract

Fluorescence guided surgery is an emerging technology that may improve accuracy of pulmonary resection for non-small cell lung cancer (NSCLC). Herein we explore optical imaging for NSCLC surgery using the well-studied protoporphyrin IX (PPIX)/5-aminiolevulinic acid (5-ALA) system. More specifically, we evaluate fluorescent patterns observed when using (1) commonly utilized *in vitro* and murine NSCLC models and with (2) spontaneous canine NSCLCs, which closely mimic human disease. Using flow cytometry and fluorescent microscopy, we confirmed that NSCLC models fluoresce after exposure to 5-ALA *in vitro*. High levels of fluorescence were similarly observed in murine tumors within 2 hours of systemic 5-ALA delivery. When evaluating this approach in spontaneous canine NSCLC, tumor fluorescence was observed in 6 of 7 canines. Tumor fluorescence, however, was heterogenous owing to intratumoral variations in cellularity and necrosis. Margin and lymph node detection was inaccurate. These data demonstrate the importance of incorporating reliable cancer models into preclinical evaluations of optical agents. Utilization of spontaneous large animal models of cancer may further provide an important intermediate in the path to human translation of optical contrast agents.

## Introduction

Non-small cell lung cancer (NSCLC) is the leading cause of cancer-related death in the United States with an estimated 164,000 patients predicted to die from the disease in 2017^1^. For patients with Stage I to Stage IIIA disease, surgical resection is the cornerstone of treatment^2^. Improved long-term survival after pulmonary resection for NSCLC is multifactorial; however, complete removal of macroscopic and microscopic disease (R0 resection) is among the most important factors^3,4^. However, even after an R0 resection, the risk of local recurrence within 5 years is up to 25%,^5,6^ suggesting that undetected residual disease remains at the surgical margin or within the ipsilateral lung.

Intraoperatively surgeons visually evaluate and palpate tissues to localize nodules, determine tumor margins, and assess for synchronous disease. Unfortunately, these visual and tactile cues are subjective, somewhat dependent on experience, and may lead to erroneous decisions resulting in local recurrences. In order to improve intraoperative cancer assessment, surgeons have traditionally relied upon frozen-section analysis. Frozen section analyses are costly, time consuming, and often inaccurate^5,7^. These deficiencies have prompted development of additional localization techniques including intraoperative CT, wire localization, intratumor dye marking, and fluoroscopy^8^. These approaches also have limitations insomuch they are time consuming, expensive, potentially dangerous, and user-dependent.

Alternatively, fluorescence guided surgery (FGS), also commonly termed intraoperative molecular imaging (IMI), is an emerging technology that provides surgeons with objective, fluorescent visualization of cancers, thus allowing for real-time disease localization, margin assessment, and lymph node identification. A number of approaches to fluorescence imaging, including affinity based approaches (agents targeting the tumor or tumor microenvironment directly) or activity based approaches (agents are enzymatically activated by tumor), have been described^9,10^. These approaches have been found safe and effective for a wide range of common malignancies including brain, ovarian, testicular, colorectal, gastric, esophageal, pancreatic, hepatocellular, renal, bladder, prostate, parathyroid, and thyroid cancers^9,10^.

Over the last decade, our group has successfully translated several FGS approaches for NSCLC to clinical trials^11–17^. In our earliest human studies, we found that near infrared (NIR) fluorescence imaging using indocyanine green (ICG) was capable of identifying primary lung tumors^11^, pulmonary metastases^18^, and mediastinal disease^19^ in canine and human patients. Although largely effective, ICG accumulates in tumors through enhanced permeability of the tumor vasculature and decreased lymphatic drainage (the “enhanced permeability and retention” effect), two non-specific mechanisms. Because of this mechanism of action, ICG is a highly sensitive drug that cannot differentiate inflammation from neoplasia. To increase specificity, our group has recently begun evaluating a NIR, folate receptor-targeted agent, OTL38^14,17^. In these initial reports, we have found that greater than 90% of pulmonary adenocarcinomas accumulate OTL38 and generate tumor fluorescence. Furthermore, we have reproducibly detected nodules as small as 3 mm. Although sensitive for pulmonary adenocarcinomas, OTL38 has unknown efficacy for other NSCLC histologies. In each of aforementioned experiences, evaluation in a spontaneous large-animal (canine) model of lung cancer provided critical toxicology, feasibility and efficacy data, which predicted human clinical trial results.

Given previous successful translation of optical imaging approaches for thoracic malignancies, we have become interested in expanding our FGS arsenal to incorporate the well-studied visual range contrast agent protoporphyrin IX (PPIX), which is a metabolite of 5-aminolevulinic acid (5-ALA). 5-ALA/PPIX has been described for fluorescent imaging or photodynamic therapy of a range of cancers, including central nervous system tumors^20–22^, gastrointestinal malignancies^23^, and genitourinary neoplasms^24^. A number other of murine tumors models have also been studied. In these studies, 5-ALA has been found safe and capable of improving complete resection^20–22^.

Using lung cancer as a model, we aim to evaluate systemic 5-ALA as FGS agent applicable to a broad range of NSCLC histologies. More specifically, we explore the feasibility of fluorescence guided surgery with 5-ALA beginning with *in vitro* NSCLC models, then *in vivo* murine models, and finally in a clinical trial involving 12 canines presenting with spontaneous lung cancers. In addition to exploring the translational potential of 5-ALA for NSCLC, this work highlights the importance of evaluating novel optical contrast agents in reliable models that recapitulate the complexity of human malignancy.

## Results

### Murine and Human Lung Cancer Models generate PPIX from 5-ALA *in vitro*

We first sought to determine if NSCLC models were capable of generating the fluorescent compound, PPIX, from 5-ALA *in vitro*. To assess this, several commonly utilized NSCLC models (TC1, LKR, A549, L55 and ChaGo-K-1) were exposed to culture media spiked with 5-ALA (1 mM) for various time periods. After incubation, cells were harvested and analyzed by flow cytometry and fluorescent microscopy.

**Figure 1:**
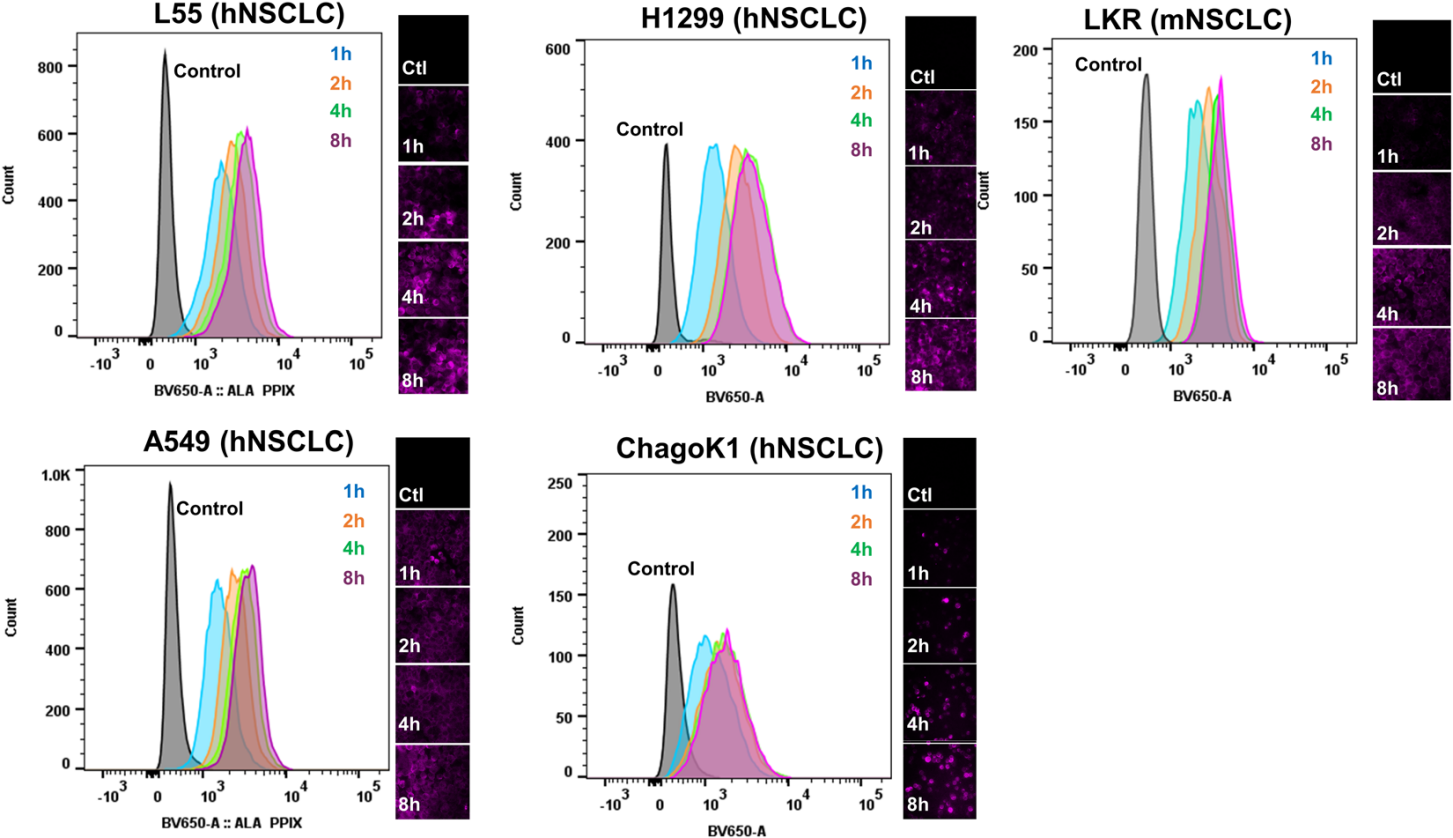
NSCLC models generate PPIX *in vitro:* After exposing NSCLC models (L55, A549, ChaGoK1, H1299, and LKR) to media spiked with 5-ALA (1 mM) for various times, fluorescence was assessed by flow cytometry and by fluorescent microscopy (using a 610 nm long pass filter). Fluorescence was observed as early as 1 hour after exposure to 5-ALA. After 4 hours, additional exposure to 5-ALA did not increase fluorescence. hNSCLC-human NSCLC model; mNSCLC-murine NSCLC model.

By flow cytometry, high levels of fluorescence were observed among all cell lines tested (Fig. 1a). We noted that fluorescent signal was appreciated in all lines after 1 hour of exposure to 5-ALA. After 4 hours, maximum fluorescence levels were achieved. Additional exposure to 5-ALA for greater than 4 hours did not increase fluorescent signal. By fluorescent microscopy, we similarly observed fluorescent signal across all models (Fig. 1b). Again, exposure to 5-ALA for greater than 4 hours did not significantly increase fluorescent signal intensity.

These results suggest that NSCLC models are capable of rapid generation the fluorescent compound, PPIX, after exposure to 5-ALA.

### NSCLC models generate PPIX from 5-ALA *in vivo*

**Figure 2:**
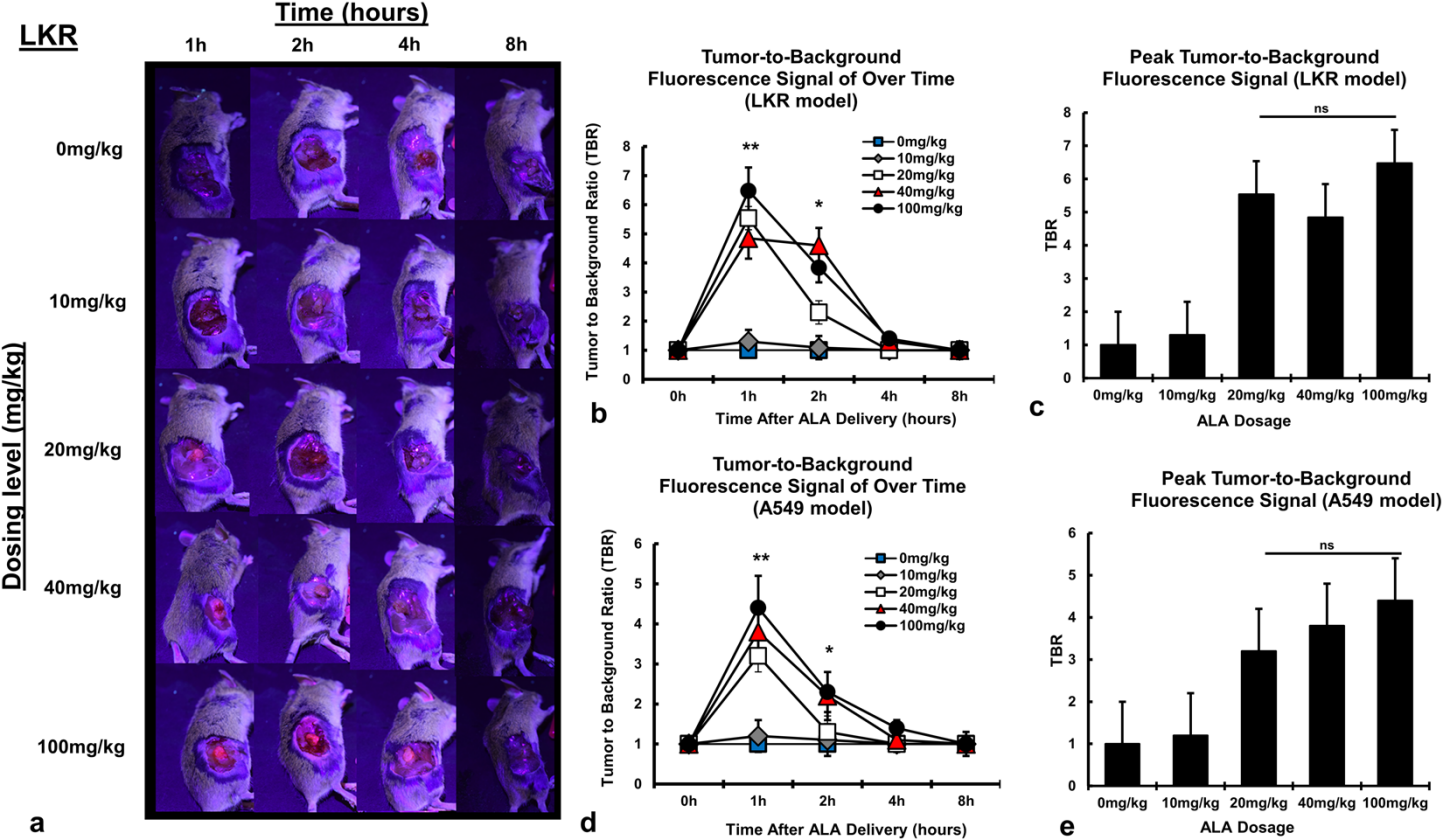
NSCLC models generate PPIX from 5-ALA *in vivo:* Mice bearing LKR flank tumors were randomized to variable doses of 5-ALA (0 mg/kg to 100 mg/kg), then imaged using the FloCam Imaging System. (**a**) Fluorescence of LKR NSCLC flank tumors was observed at higher dosing levels for up to 2 hours. (**b**) TBR of LKR flank tumors was calculated point and plotted over time. (**c**) Peak TBRs for LKR tumors were compared for dosing levels; of note, peak TBR for each concentration was observed 1 hour after drug delivery. The previous experiments were repeating in mice bearing A549 NSCLC xerographs, and the (**d**) TBR of A549 flank tumors was calculated point and plotted over time. (**e**) Peak TBRs for A549 tumors were compared for dosing levels. *p < 0.05, **p < 0.01, ns—not significant.

Given *in vitro* data suggesting that NSCLC models generate PPIX, we next sought to evaluate fluorescence *in vivo*. Mice bearing LKR flank tumors were randomized to oral 5-ALA at 5 dosing levels (n = 3 per group). Mice were imaged using the FloCam at various time points. In mice receiving the lowest dosing (10 mg/kg), negligible fluorescent signal was observed within tumors (Fig. 2a). At higher dosing levels (20 mg/kg, 40 mg/kg and 100 mg/kg), peak TBR fluorescence was observed at 1 hour following delivery (Fig. 2b). Interestingly, peak TBRs were similar among those mice randomized to these dosing levels; p > 0.05 (Fig. 2c). Despite similar peak TBRs at 1 hour, tumors in mice randomized to the two highest dosing levels were associated with higher signal at later time points; p = 0.01 (Fig. 2b).

When repeating these experiments in mice bearing A549 tumors, similar results were observed. As can be seen in Fig. 2d, peak TBR in A549 tumors was observed 1 hour following drug delivery. Tumor specific signal rapidly degraded, and only persisted in mice randomized to the two highest dosing levels (Fig. 2d). Lastly, as in mice bearing LKR tumors, peak TBRs among the higher dosing levels were similar (Fig. 2e).

These data suggest that NSCLC models are capable of generating PPIX *in vivo*. Further, these data suggest that dramatic increases in 5-ALA dosing likely do not impact peak TBR; however, increased dosing levels may increase the time which tumors display fluorescence.

### Evaluation of Intraoperative Fluorescence Imaging Using 5-ALA a Large Animal Model of Lung Cancer

Given encouraging results in *in vitro* and *in vivo* models, we sought to evaluate this approach in a reliable large animal model of pulmonary malignancy.

### Study Population

Between September 2015 and February 2017, 12 canines with suspected primary pulmonary tumors were deemed surgical candidates and were recruited from the University of Pennsylvania School of Veterinary Medicine. The most common breed was a mixed breed (n = 3, 25%), followed by and 8 other breeds including 2 Weimaraners (16.7%), an Ibizan (8.3%), a Mastiff, a Jack Russell Terrier (8.3%), a Greyhound (8.3%), a Bichon Terrier (8.3%), a Staffordshire Terrier (8.3%), and a Labrador Retriever (8.3%). Eleven (91%) males and one (9%) female included in the trial. The median age was 12 years (IQR, 10 to 13 years), and median weight was 29.4 kg (IQR, 7.8 to 35.5 kg). All tumors were subpleural and were within 2 cm of the pleural surface as determined by preoperative CT.

Seven canines had primary pulmonary tumors (58.3%), including 4 adenocarcinomas, two carcinomas, and one papillary carcinoma. Four canines had non-pulmonary tumors, which including histiocytic sarcoma (n = 1), metastatic mammary carcinoma (n = 1), neuroendocrine tumor (n = 1), and mast cell tumor (n = 1). One canine (Subject 7) was euthanized during surgery because the lung tumor was unresectable (the owners did not permit biopsy or necropsy in this case).

A full summary of the 12 included canines is provided in Table 1.

**Table 1:** Clinical and histopathologic data of 12 canine subjects.

ID	Breed	Age (years)	Weight (kg)	Gender	Tumor Location	Maximum diameter (cm)	Pathology	Adverse Event	Fluorescent (yes/no)	TBR
1	Ibizan	11	30.4	Male	Right Cranial	5.0	pulmonary adenocarcinoma	nausea during drug delivery	yes	1.8
2	Mixed Breed	13	6.3	Male	Right Caudal	4.0	pulmonary adenocarcinoma	none	no	1.4
3	Mastiff	9	45.0	Female	Left Caudal	3.0	metastatic mammary	none	yes***	1.5
4	Mixed Breed	15	11.0	Male	Right Caudal	6.7	pulmonary carcinoma	none	yes	1.8
5	Weimaraner	12	44.7	Male	Right Cranial	12	papillary carcinoma	none	yes	2.2
6	Weimaraner	12	31.5	Male	Left Caudal	5.0	pulmonary carcinoma	none	yes	2.3
7	Jack Russell Terrier	15	7.3	Male	Left Caudal	unresectable*	unknown**	none	no	1.0
8	Greyhound	6	33.6	Male	Left Cranial	2.7	histiocytic sarcoma	none	yes	1.5
9	Bichon	13	7.0	Male	Right Middle	6.0	pulmonary adenocarcinoma	none	yes	2.2
10	Staffordshire Terrier	10	28.5	Male	Left Caudal	4.0	neuroendocrine tumor	none	no	1.0
11	Labrador Retriever	10	37.3	Male	Right Middle	5.6	mast cell tumor	none	no	1.3
12	Mixed Breed	13	8.4	Male	Left Caudal	5.6	pulmonary adenocarcinoma	none	yes	2.1

TBR - *in situ* tumor to fluorescence background ratio, *too large for resection thus no measurements obtained, **canine euthanized before resection thus no pathology obtained, ***mass displayed fluorescence only upon *ex vivo* tumor bisection.

### Safety and Toxicity

One canine (Subject 1, Ibizan) experienced mild nausea immediately following delivery of 5-ALA. No associated physical examination or biochemical abnormalities were noted. Symptoms resolved after 10 minutes of observation. In all surviving canines, no intraoperative or postoperative toxicity was noted after at minimum of 6 months of follow-up.

### *In vivo* fluorescence of Lung Tumors

**Figure 3:**
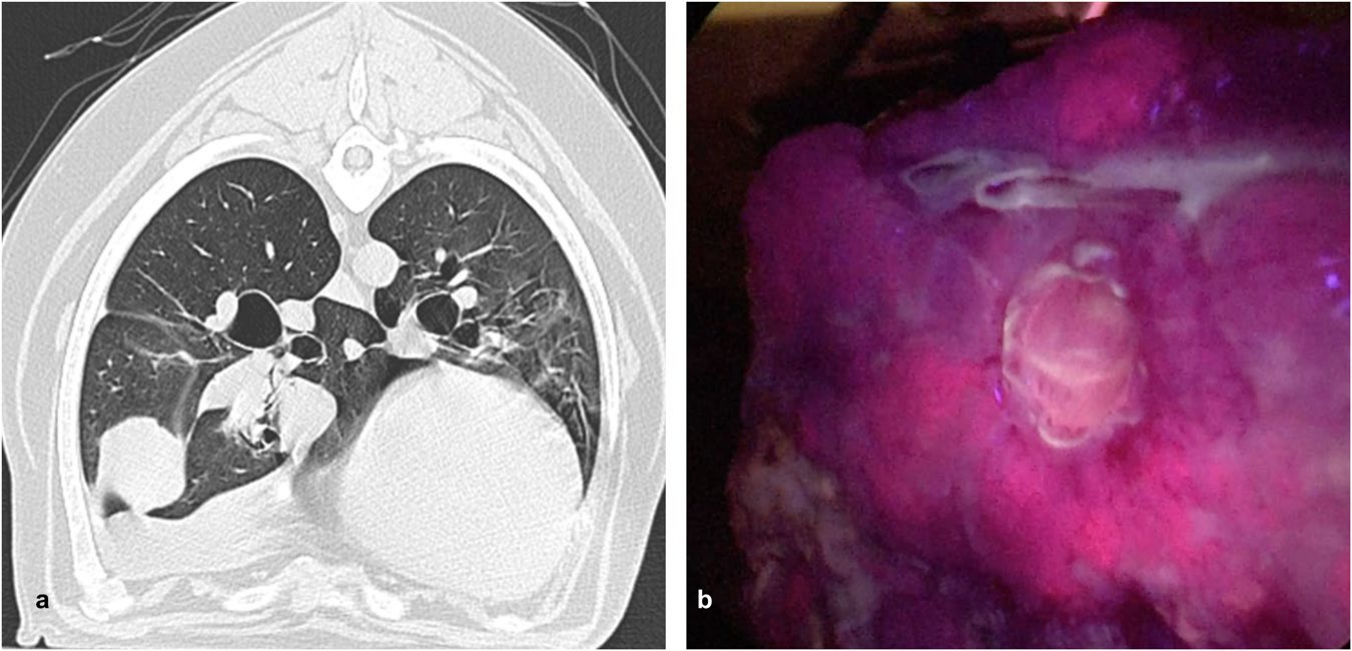
Representative example canine tumor displaying *in vivo* PPIX fluorescence during pulmonary resection: Canine 5 was a 12-year-old male, Weimaraner that presented with a 12 cm papillary carcinoma of the right cranial lobe (**a**). During pulmonary resection, the tumor displayed observable levels of fluorescence, with a TBR of 2.2 (**b**).

Six of the seven primary pulmonary tumors displayed *in situ* fluorescence during resection while 1 displayed no fluorescence signal (Fig. 3). Tumor fluorescence was only uniform in 2/6 canines; in the other 4 animals, surface fluorescence was irregular. The median Tumor to Background Fluorescent Ratio (TBR) of fluorescent tumors was 2.1 (IQR, 1.8 to 2.2); the TBR for the non-fluorescent tumor was 1.4. Of the remaining non-pulmonary tumors, only the histiocytic sarcoma displayed fluorescence (TBR = 1.5). The remaining dogs had no visible tumor fluorescence (TBR 0.9–1.3). Upon histologic correlative analysis, tumors displaying fluorescence heterogeneity were found to be marked with patchy necrosis.

**Figure 4:**
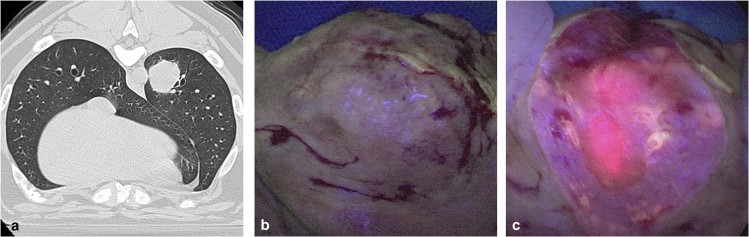
Example canine tumor displaying fluorescence only upon *ex vivo* tumor bisection: Canine 3 was a 9-year-old male, Mastiff that presented with a 3 cm metastatic mammary tumor of the left caudal lobe (**a**). During pulmonary resection, the tumor displayed no fluorescence (**b**). However, upon tumor bisection fluorescence was noted, with a TBR of 2.8 (**c**).

Two tumors (16.7%), including one adenocarcinoma and the metastatic mammary tumor, displayed increased fluorescence upon tumor bisection (TBR 2.4 and 2.8 respectively -Fig. 4) Of note, fluorescence upon tumor bisection was higher than *in situ* fluorescence levels (p = 0.01). Upon gross evaluation, areas of highest fluorescence correlated to highly cellular and viable portions of the tumor.

### Clinical Impact of fluorescent guided surgery using 5-ALA/PPIX

**Figure 5:**
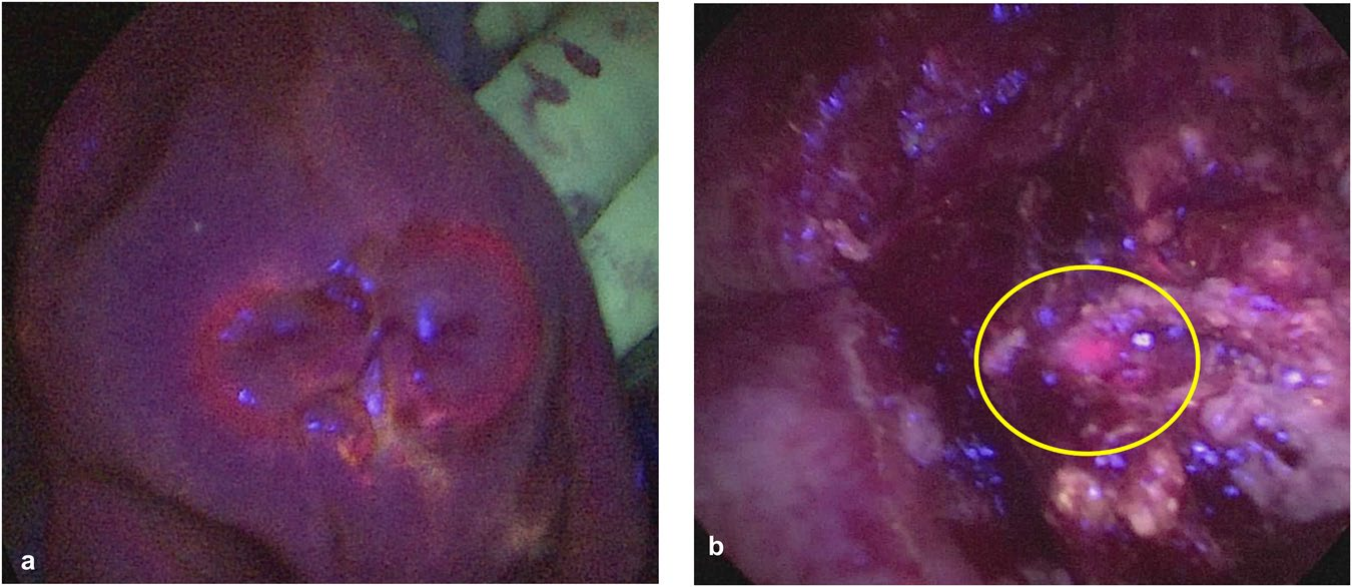
Margin assessment during fluorescent imaging: Canine 8: Consistent fluorescence was observed at tumor margin (**a**). Canine 4: Inconsistent fluorescence was observed at a grossly positive margin (**b**). yellow circle—fluorescent signal in pink.

Fluorescence guided surgery has been developed to improve a surgical oncologist’s ability to accomplish several intraoperative tasks including margin assessment, determination of lymph node involvement, and identification of synchronous cancers. Of the 7 canines with primary pulmonary tumors, fluorescence imaging identified peripheral tumor margins in 2 (29%) (Fig. 5a). In the remaining 5 (71%) cases, tumors either displayed a lack of fluorescence (n = 1) or unreliable fluorescence patterns that did not extend to the gross tumor margins (Fig. 5b).

Regional bronchial lymph node packets were harvested in 4 of the 7 (57%) primary pulmonary tumors. Of these harvested lymph nodes, one was positive for micrometastatic disease on final pathology. This node did not fluoresce during fluorescent imaging. Of the 3 pathologically clear lymph node samples, one displayed high levels of fluorescence. Although these numbers are small, the observed accuracy rate fluorescence was 50% (1 false-negative, 1 false-positive, and 2 true-negatives).

Lastly, in this cohort, no synchronous cancers were identified by fluorescent imaging. However, on final pathologic review specimen, no synchronous diseases were found.

Taken together, these results suggest that the addition of 5-ALA to standard pulmonary resection is not a consistent way to add accurate information that impact intraoperative decision making.

### *In Situ* Fluorescence is predicted by

**Figure 6:**
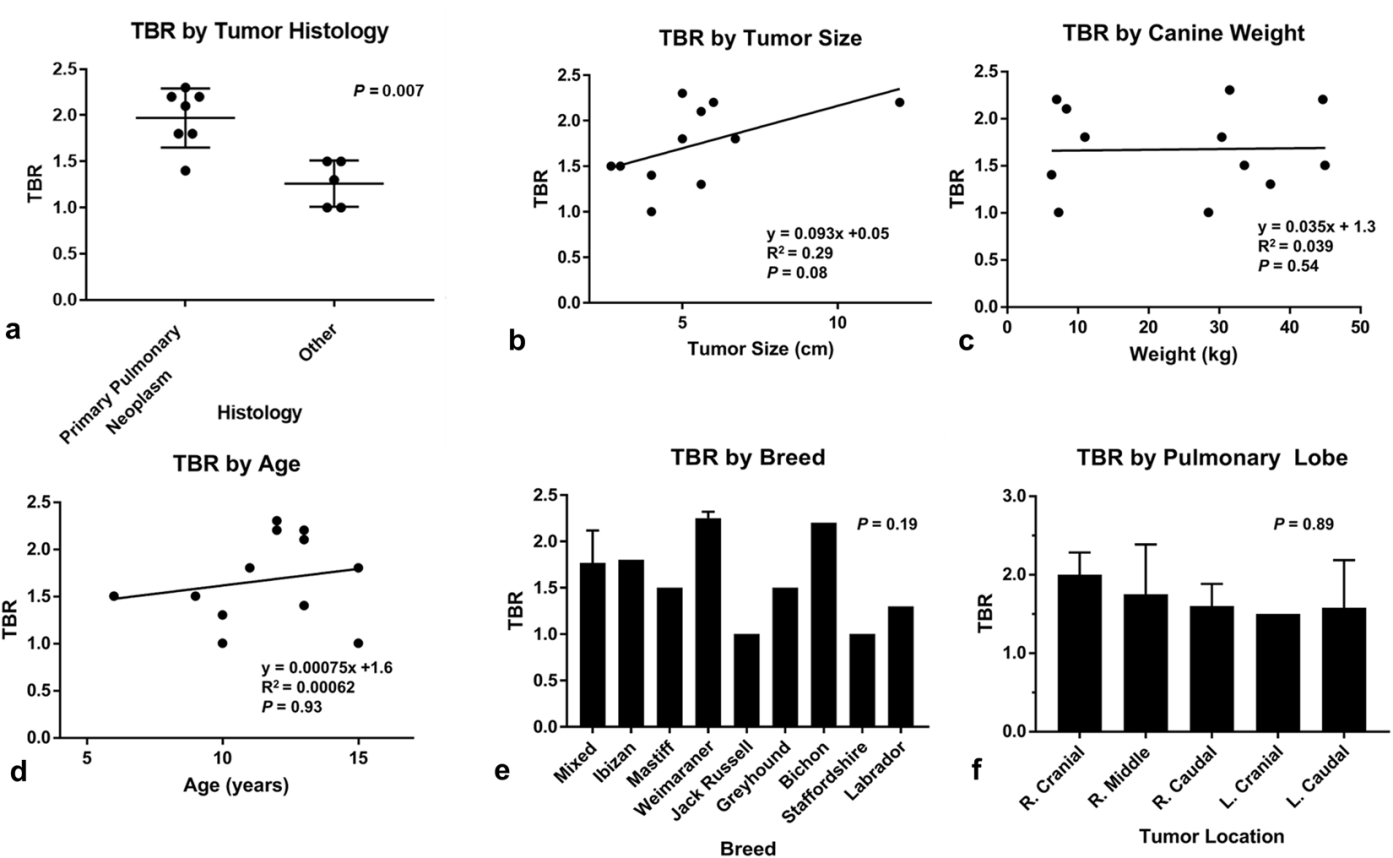
Clinical and Histopathologic Variables to Predict *in situ* TBR: (**a**) TBR as a function histology, (**b**) TBR as a function tumor size, (**c**) TBR as a function of canine weight, (**d**) TBR as a function of canine age, (**e**) TBR by tumor canine breed, (**f**) TBR by tumor location with anastomotic pulmonary lobe. Results are expressed as mean, standard deviation.

To better predict potential utility of fluorescence guided surgery with 5-ALA, several clinically and histopathologically relevant factors were evaluated in effort to determine influence on *in situ* tumor fluorescence (Fig. 6). Age, breed, gender, tumor location, tumor size, histologic subtype were chosen as this information is routinely available to surgeons prior to resection. We found that, in general, primary pulmonary tumors were more fluorescent than other tumors (Fig. 6a). We appreciated trends toward increased fluorescence with increasing tumor size; however, this did not approach statistical significance (p = 0.08) (Fig. 6b). Other evaluated variables poorly predicted fluorescence (Fig. 6c–f). These data, unfortunately, suggest that fluorescence patterns are largely unpredictable based on the information that is commonly available to surgeons preoperatively.

## Discussion

Fluorescence guided surgery using the 5-ALA/PPIX system has proven successful in improving resection for several tumor types^20–24^. In this study, we explore this approach’s ability to improve detection of NSCLC at the time of oncologic pulmonary resection. We found that common *in vitro* and murine NSCLC models reproducibly generate PPIX after exposure to 5-ALA.When evaluating this approach in a canine model that recapitulates human NSCLC, 6 of 7 of tumors displayed fluorescence. Fluorescence in general was heterogenous and failed to improve the surgeon’s ability to identify tumor margins or metastatic lymph nodes. The discrepancy between *in vitro*/murine data with canine data highlights the importance of evaluating optical contrast agents in cancer models that closely recapitulate human malignancy.

The fluorescent compound PPIX is a product of ALA metabolism. Tumor identification using 5-ALA-based imaging approaches thus rely on increased accumulation of PPIX in tumors after administration of 5-ALA. Several studies have demonstrated that selective PPIX accumulation within neoplastic cells occurs due to enzymatic alterations in the hemoglobin metabolism pathway. One such alteration is an increased activity of porphobilinogen deaminase, an enzyme which plays a role in ALA breakdown^25^. Additional reports have described decreased activity of ferrochelatase, an enzyme which metabolizes PPIX to hemoglobin^26,27^. Together, these enzymatic changes result in the increased accumulation of PPIX in epithelial malignancies.

Although we did not directly evaluate enzymatic levels within tumor models, we demonstrate that preclinical syngeneic and xenograft NSCLC models accumulate PPIX after exposure to 5-ALA. We found this both *in vitro* and *in vivo* scenarios. Similar to other reports, the optimal time for fluorescent evaluation ranged from 1–4 hours^28^. Although commonly used for preclinical optical contrast agent evaluation, there are obvious limitations that render *in vitro* and murine models poor representations of human NSCLC. Most obviously, such models fail to reproduce the complex biology associated with human cancers, including NSCLC. Human and canine malignancies are complex biologic systems, consisting of intricate stromal networks, infiltrating leukocytes, and polyclonal tumor cell variants. *In vitro* and murine models, however, are artificial systems which arise from monoclonal tumor cell expansion. As such, efficacy in these controlled scenarios does not necessarily equate to success in humans.

With these limitations and the high costs associated with human translation in mind, our group has incorporated preclinical drug evaluations in canines presenting with spontaneous pulmonary malignancies into our translational pathway^11,14,29^. In fact, over the last decade, we have utilized this approach during evaluation of two near-infrared molecular imaging probes (ICG and a folate receptor -targeted probe called OTL38). In each circumstance, we found that canine results closely resemble human outcomes^11,13,14,17,18,29^.

It is well established that canines develop lung cancers based on the same carcinogenic and physiologic factors as humans. Furthermore, canines have a diverse genetic background similar to the heterogeneity observed in the human population. Unlike murine models which can fail to replicate the biodistribution and pharmacokinetics of the human, the scale of the dog model provides a reliable opportunity for better understanding of probe pharmacodynamics for translation to human clinic trial.

To this end, after obtaining encouraging *in vitro* and murine data pertaining to 5-ALA, we explored fluorescence guided surgery in a large animal clinical trial involving 12 canines that presented with spontaneous pulmonary malignancies. We appreciated excellent safety and found that 6 or 7 primary lung tumors displayed *in vivo* fluorescence. Only 1 of 5 non-pulmonary tumors displayed signal. Of the 6 fluorescent pulmonary tumors, fluorescence was patchy and non-uniform. We feel that these inconsistencies are partially related to the prevalence of heterogenous patterns of necrosis observed in pulmonary tumors of this size. Owing to variable fluorescent patterns, we found FGS with 5-ALA unreliable in identifying tumor margins, inaccurate in lymph node detection, and of little utility when evaluating for synchronous malignancies. Furthermore, we found fluorescence patterns unpredictable based on clinical and histopathologic information available to surgeons preoperatively.

These large animal data highlight several limitations of fluorescence guided surgery of pulmonary neoplasms using 5-ALA. These limitations would otherwise have been overlooked if preclinical evaluation only incorporated more rudimentary *in vitro* and murine NSCLC models. For example, PPIX accumulated in the neoplastic cells requires excitation at wavelengths of 405 nm and fluoresces with a major emission peak at 634 nm. Utilization of visible range fluorophores are significantly limited due to poor tissue penetration and significant autofluorescence of normal tissues^30,31^. These limitations become obvious hurdles when evaluating tumors with deep tissue planes and irregular margins. In canines, this likely explains the observed inconsistent margin assessment and the lack of identification of synchronous disease that has been described for near infrared agents^12,19^. We point out that current success of 5-ALA/PPIX in human glioma patients is likely a result of glioma’s histology which is marked by dense cellularity and high vascularization. Nevertheless, the lack of penetration and autofluorescence limits 5-ALA/PPIX in the context of central nervous system tumors, which is a primary impetus for the exploration of NIR imaging probes for neurologic malignancies (eg, NCT03510208).

Next, evaluation in a canine trial animal model allowed us to better gauge utility of 5-ALA/PPIX in identifying bronchial lymph node involvement. In this study, fluorescent imaging correctly identified two tumor negative lymph nodes; however, it also yielded one false-positive and one false-negative result in two other cases (50% accuracy). Although these are low numbers, these results are nonetheless disappointing as accurate identification of tumor positive lymph nodes and tumor margin detection are the two most significant intraoperative challenges in surgery for NSCLC. Again, data pertaining to lymph node assessment is difficult, if not impossible, to capture using more traditional murine models of metastases which involving flank tumors followed by systemic tumor cell delivery by venous injection^32^.

The results of this study are disappointing given the seemingly specific accumulation of protoporphyrin IX in a broad range of tumors^20–24^. The lack of uniform fluorescence, inaccurate margin assessment and lymph node evaluation seen with ALA in this canine model compare unfavorably with our previous studies involving the NIR dyes, ICG and OTL38^11,14^. In these studies, NIR fluorescence using ICG correctly identified 100% of pulmonary tumors^11^, while NIR fluorescence with OTL38 correctly identified 100% pulmonary malignancies and tumor margins, and correctly identified tumor positive lymph nodes in several cases^14^. Given these results, our investigations of 5-ALA for surgical navigation during human pulmonary resection have been halted.

In summary, the presented data provide information demonstrating that *in vitro* and murine models of NSCLC reliably generate the fluorescent compound, PPIX, after exposure to 5-ALA. However, in reliable canine models of NSCLC which closely mimic human disease, fluorescence guided surgery using 5-ALA/PPIX fails to demonstrate utility. These results suggest that alternative optical contrast agents should be explored to further enhance FGS for pulmonary malignancies. More importantly, these data demonstrate the importance of incorporating reliable cancer models into the preclinical evaluation of novel optical imaging agents before translation to early phase human trials.

## Methods

### Cell Lines and Small Animal Models of Lung Cancer

The spontaneously metastatic murine lung cancer line, LKR, was derived from an explanted pulmonary tumor from an activated KrasG12D mutant mouse that had been induced in an F1 hybrid of 129 Sv.J and C57BL/6^33^. The murine lung cancer cell line, TC1, was derived from mouse lung epithelial cells immortalized with HPV-16 E6 and E7 and transformed with the c-Ha-ras oncogene^34^. A549, H1299 and ChaGo-K-1 are commercially available human NSCLC models. All lines were cultured and maintained in media consisting of RPMI (Mediatech, Washington, DC) supplemented with 10% fetal bovine serum (FBS; Georgia Biotechnology, Atlanta, GA), 5 Ag/mL penicillin/streptomycin, and 2 mmol/L glutamine.

The Institutional Animal Care and Use Committees of the University of approved all study murine protocols described in this publication, and experiments were conducted in compliance with the Guide for the Care and Use of Laboratory Animals. Female B6x129/J1 hybrid (6–8 weeks old) mice were purchased from Charles River Laboratories, Inc. (Wilmington, MA). All mice were maintained in a pathogen-free animal facility for one week prior to experimentation. Mice were injected subcutaneously on the flank with 2 × 10^6^ LKR cells (suspended 100 μL PBS).

### Reagents

5-aminolevulinic acid HCL (5-ALA) was provided by medac GmbH (Hamburg Germany). For *in vitro* studies, 5-ALA was dissolved in media (see below) at a concentration of 1 mM. For *in vivo* murine studies, 5-ALA was suspended in 0.9% normal saline and administered to mice by oral gavage at varying concentrations. Synthetic PPIX at was purchased from Sigma-Adlrich (St. Louis, MO, USA), and used as a positive control by dissolving in 2.5 mM NaOH.

### *In Vitro* Production of PPIX by flow cytometry and fluorescent microscopy

Cell lines were seated overnight in 6-well plates at a concentration 1 × 10^6^ cells per plate. After resting cells overnight, plated cells were exposed to media spiked with 5-ALA (1 mM) for various times. Cellular conversion of 5-ALA to PPIX was assessed by flow cytometry using an LSR Fortessa (BD Biosciences, San Jose, CA, USA), specifically utilizing the violet laser (excitation 405 nm) and fluorescent emission between 660 to 760 nm. Fluorescence was also assessed using a fluorescent microscope equipped with an excitation light of 395/25 nm (Lumencor, Inc., Beavertown, OR, USA) with PPIX-specific emission being captured by a 610nm-long-pass filter (Chroma, Bellow Falls, VT).

### Fluorescence Quantification Using a Small Animal Optical Imaging System

The “FloCam” is a digital imaging system based on a dual CCD camera system previously described^35^ (BioVision Technologies Inc, Exeter, PA, USA). The system uses two QIClick™ digital CCD cameras from QImaging (British Columbia, Canada), one for white brightfield and one for fluorescence overlay. The light source is a Spectra X Light Engine (Lumencor, Inc., Beavertown, OR, USA). For the work described, an excitation light of 395/25 nm and fluorescence was selected for by using a 610 nm long pass filter (Chroma, Bellow Falls, VT, USA).

### Large Animal Trial of ALA-Derived Protoporphyrin IX

This study was approved by the Institutional Animal Care and Use Committee of the University of Pennsylvania. Between September 2015 and February 2017, 12 consecutive canines with suspected primary lung tumors that were deemed surgical candidates were recruited from the University of Pennsylvania School of Veterinary Medicine. Written informed consent was obtained from each owner and the consent document was approved by the Veterinary School’s Privately Owned Animal Protocol Committee. Sample size selection was based on our group’s previous experiences with NIR and folate receptor-targeted IMI^18,36^. 5-ALA (20 mg/kg) was administered two to four hours prior to surgery. Maropitant citrate (1 mg/kg IV) was administered three hours before surgery to prevent nausea from ALA administration. Of note, because these were client owned animals, higher doses of 5-ALA were not evaluated given a paucity of safety data involving dosing ranges above 20–25 mg/kg.

During resection, surgeons utilized white-light (bright-field imaging) and finger palpation through a standard-of-care thoracotomy to confirm known nodules. Additionally, surgeons evaluated the remainder of the thorax using standard white-light thoracoscopy to assess for synchronous disease. Next, fluorescent imaging using a Karl-Storz endoscopic system optimized for 5-ALA/PPIX fluorescence (Karl Storz GmbH & Co. KG Tuttlingen, Germany) was used to inspect the ipsilateral hemithorax. Fluorescence was assessed from the tumor and normal parenchyma in the affected lobe or, in the case of large tumors, grossly normal lung in ipsilateral lobes. Tumor margins were imaged in 4 radial directions, designated the 12, 3, 6, and 9 o’clock positions. Grossly normal lung 5 mm and 10 mm extending away from the tumor periphery was then imaged where applicable.

Following pulmonary resection, the remaining hilus and visible bronchial lymph nodes were imaged. All lymph nodes that were visualized and removed were part of lymph node stations that would have otherwise been removed as part of standard of care. Excised specimens were re-imaged *ex vivo* prior to submitting them for histopathological evaluation. All tissues were then fixed in 10% formalin, embedded in paraffin, sectioned, and evaluated by a veterinary pathologist.

### Fluorescence Quantification and Statistics

*Post hoc* fluorescence was quantified using ImageJ (http://rsb.info.nih.gov/ij) region of interest (ROI) software to select the entire tumor. A 2–3 cm representative section of normal lung was also selected, and tumor-to-background fluorescence ratios (TBRs) were calculated for all identified lesions. At least two surgeons confirmed ROIs. Given the small sample size, data are presented as median (IQR) unless noted. Differences between groups were compared by the Mann-Whitney test (two groups) or Kuskal-Wallis test (three or more groups). Correlation among two continuous variables were assessed using linear regression. Statistics were generated using Stata Statistical Software: Release 14 (College Station, TX: StataCorp LP). A p-value of 0.05 or less was considered statistically significant.
